# Association of moderate-to-vigorous physical activity with neck circumference in eight Latin American countries

**DOI:** 10.1186/s12889-019-7153-y

**Published:** 2019-06-24

**Authors:** Gerson Luis de Moraes Ferrari, Irina Kovalskys, Mauro Fisberg, Georgina Gomez, Attilio Rigotti, Lilia Yadira Cortés Sanabria, Martha Cecilia Yépez García, Rossina Gabriella Pareja Torres, Marianella Herrera-Cuenca, Ioná Zalcman Zimberg, Viviana Guajardo, Michael Pratt, Carlos Pires, Dirceu Solé, on behalf of the ELANS Study Group, Luis A. Moreno, Beate Lloyd, Brenda Lynch, Mariela Jauregui, Alejandra Guidi, Luis Costa, Regina Mara Fisberg, Mauro Fisberg, Irina Kovalskys, Georgina Gómez Salas, Attilio Rigotti, Lilia Yadira Cortés Sanabria, Martha Cecilia Yépez García, Rossina Gabriella Pareja Torres, Marianella Herrera-Cuenca, Berthold Koletzko, Michael Pratt, Katherine L. Tucker, Viviana Guajardo, Ioná Zalcman Zimberg, María Paz Amigo, Ximena Janezic, Fernando Cardini, Myriam Echeverry, Natasha Aparecida Grande de França, Guadalupe Echeverría, Leslie Landaeta, Óscar Castillo, Luz Nayibe Vargas, Luisa Fernanda Tobar, Yuri Milena Castillo, Rafael Monge Rojas, Anne Chinnock, María Elisa Herrera Fontana, Mónica Villar Cáceres, María Belén Ocampo, Rossina Pareja Torres, María Reyna Liria, Krysty Meza, Mellisa Abad, Mary Penny, Maritza Landaeta, Betty Méndez, Maura Vasquez, Omaira Rivas, Carmen Meza, Servando Ruiz, Guillermo Ramirez, Pablo Hernández, Alexandre D. P. Chiavegatto Filho, Priscila Bezerra Gonçalves, Claudia Alberico, Gerson Luis de Moraes Ferrari

**Affiliations:** 1 Centro de Investigación en Fisiologia del Ejercicio-CIFE, Universidad Mayor, José Toribio Medina, 29. Estacion Central, Santiago, Chile; 2 Disciplina de Alergia, Imunologia Clínica e Reumatologia do Departamento de Pediatria, da Universidade Federal de São Paulo, São Paulo, Brazil; 3 Commitee of Nutrition and WellbeingInternational Life Science Institute, Buenos Aires, Argentina; 4 Instituto Pensi, Fundação José Luiz Egydio Setubal, Hospital Infantil Sabará, São Paulo, Brazil; 5 Departamento de Pediatria, da Universidade Federal de São Paulo, São Paulo, Brazil; 6 Departamento de Bioquímica, Escuela de Medicina, Universidad de Costa Rica, San José, Costa Rica; 7 Centro de Nutrición Molecular y Enfermedades Crónicas, Departamento de Nutrición, Diabetes y Metabolismo, Escuela de Medicina, Pontificia Universidad Católica, Santiago, Chile; 8 Departamento de Nutrición y Bioquímica, Pontificia Universidad Javeriana, Bogotá, Colombia; 9 Colégio de Ciencias de la Salud, Universidad San Francisco de Quito, Quito, Ecuador; 10 Instituto de Investigación Nutricional, La Molina, Lima, Peru; 11 Centro de Estudios del Desarrollo, Universidad Central de Venezuela/Fundación Bengoa, Caracas, Venezuela; 12 Departamento de Psicobiologia, Universidade Federal de São Paulo, São Paulo, Brazil; 13 Institute for Public Health, University of California San Diego, La Jolla, CA USA; 14 Centre for Mathematics of the University of Trás-os-Montes e Alto Douro (CM-UTAD), Vila Real, Portugal

**Keywords:** Physical activity, Accelerometer, Neck circumference, Obesity, Body composition, Anthropometric

## Abstract

**Background:**

Physical activity is a cornerstone in the prevention and treatment of obesity. There are relatively few studies that explore the effect of accelerometer-determined moderate-to-vigorous physical activity (MVPA) on neck circumference (NC), most of them confined to single high-income countries. The present study investigated the association of accelerometer-determined MVPA with NC in adolescents and adults from eight Latin American countries, which are mostly upper-middle income countries.

**Methods:**

The sample consisted of 2370 participants (47.8% male) from the Latin American Study of Nutrition and Health, a multicenter cross-sectional nutrition and health surveillance study of a nationally representative sample from eight Latin American countries (Argentina, Brazil, Chile, Colombia, Costa Rica, Ecuador, Peru, and Venezuela). Times (min/day) in MVPA (defined as time accumulated at ≥1952 activity counts/min) was assessed by ActiGraph GT3X+ accelerometer over 7 days. NC for adolescent was categorized as abnormal if circumference was > 34.5 cm for boys and > 31.25 for girls, whereas for adults the cut-off points for abnormal were > 39 cm for men and > 35 cm women. Multilevel logistic models, including country and region as random effects and adjusted for sex, age, socioeconomic level, and educational level, were used to study the association between MVPA and NC**.**

**Results:**

The average time of MVPA was 34.88 min/day, ranging from 31.16 in Venezuela to 40.27 in Chile. Concerning NC, 37.0% of the sample was classified as having elevated NC. Chile was the country with the highest percentage of people with elevated NC (56.9%), and Colombia had the lowest percentage (24.8%). Overall, the MVPA (min/day) was associated with elevated NC (OR = 0.994, CI95% = 0.990–0.998). In Costa Rica and Peru, there were significant associations between MVPA and NC when analyzed by country.

**Conclusions:**

The present study provided evidence of significant associations between MVPA and NC in adolescents and adults from Latin America, independent of sex, age, socioeconomic level, and educational level. This analysis of accelerometry data and NC represents the first examination of these associations in eight Latin America countries. Further research is required to understand the differences between countries in the observed associations.

**Trial registration:**

ClinicalTrials.Gov NCT02226627. Retrospectively registered on August 27, 2014.

## Background

The prevalence of overweight and obesity is a major global health concern [1] that now extends beyond high-income nations to low- and middle- income nations [2]. Obesity is positively associated with developing several chronic disorders, including cardiovascular disease (CVD; i.e., type II diabetes, insulin resistance, hypertension, and cholesterol levels), mortality and life expectancy, and can be reduced by adherence to a healthy lifestyle [3–6]. Consequently, early detection of weight gain is crucial to preventing adverse long-term effects on individuals’ lifestyle [5].

Central, visceral or subcutaneous fat, is strongly associated with the risk of developing several chronic disorders. It has been discussed in the literature that other deposits of fat may also contribute to the CVD [7]. Neck circumference (NC) has been associated with waist circumference (WC), body mass index (BMI) and with components of metabolic syndrome [8–12]. NC is a simple and valid measure of fat mass, more practicable and may have better association with triglycerides, HOMA, HDL-C than BMI and WC [9].

Physical activity, a cornerstone in the prevention and treatment of obesity and CVD, has been shown to be inversely related to fatness [13, 14]. The prevalence of physical inactivity in Latin America (LA) was reported the highest worldwide [15] and ranked fifth as a risk factor for mortality in the Southern Cone of LA [16]. LA is the most urbanized region in the world (80% of its population lives in cities) [17] and has rising prevalence of obesity, chronic diseases, and physical inactivity [18, 19]. Obesity and physical inactivity are now especially important public health challenges in LA [20].

The objective assessment of moderate-to-vigorous physical activity (MVPA) using accelerometers has become more common practice in research originating from high-income countries [14, 21]. In contrast, given the cost associated with using objective measures like accelerometers and the lack of research in this field in most LA countries [22], there are relatively few studies using this technology in LA countries [23]. To our knowledge, there has been no examination of the association between objective assessment of MVPA using accelerometers with NC in a nationally representative sample from LA. Thus, the purpose of this article is to investigate the association of accelerometer-determined MVPA with NC in adolescents and adults from eight LA countries.

## Methods

Study design and participants.

The Latin American Study of Nutrition and Health (*Estudio Latinoamericano de Nutrición y Salud*; ELANS) is a cross-sectional, multinational representative sample conducted in eight LA countries representing a total of 9218 individuals, aged 15–65 years, stratified by geographical location, gender, age, and socioeconomic level (SEL). The overarching ELANS protocol was approved by the Western Institutional Review Board (#20140605) and is registered at Clinical Trials (#NCT02226627). Additional site-specific protocols were also approved by the ethical review boards of the participating institutions. All participants provided informed consent/assent for participation in their country-level study. The rationale and design of the study are available in more detail elsewhere [24].

### Accelerometer

The Actigraph GT3 × + accelerometer (ActiGraph, Ft. Walton Beach, United States) was used to objectively monitor MVPA in 29.6% of the total sample. The accelerometer was worn around their waist on a belt for 7 consecutive days and the participants were instructed to wear the instrument.

Participants were encouraged to wear the accelerometer at least 12 h/day for at least 7 days of wear time following the removal of sleep, showering or swimming time [25, 26]. The minimal amount of accelerometer data that was considered acceptable for analytical purposes was 5 days (including at least one weekend day) with at least 10 h/day of waking wear time. After exclusion of the nocturnal sleep period time, waking nonwear time was defined as any sequence of at least 60 consecutive minutes of zero activity counts. Data were collected at a sampling rate of 30 Hz and downloaded in periods of 60 s [27].

On the eighth day of data collection, the research team went to the house of the participants to remove accelerometers. The research team confirmed that the data were completed using the latest version of Actilife software (version 6.0; ActiGraph, Pensacola, FL) available.

Activity cut-points were based on Freedson et al. [28] for counts/min. The cut-points capture the sporadic nature of activity and provide the best classification accuracy among the currently available cut-points for physical activity. Specifically, moderate physical activity was defined as time accumulated at ≥1952–5724 activity counts/min, ≥5725 activity counts/min for vigorous physical activity, and ≥ 1952 activity counts/min for MVPA [28] not including the sleep period and non-wear time. The Actigraph cut-points that were used in the present study were derived from studies of a previous model of the Actigraph [29–31]. Reliability and validity of accelerometers have been documented extensively [29–31].

### Neck circumference

NC (in centimeters) was measured at the point just below the larynx (thyroid cartilage) and perpendicular to the long axis of the neck (with the tape line in the front of the neck at the same height as the tape line at the back of the neck) using an inelastic tape measure [32]. Each measurement was repeated twice to ensure accuracy, and the average was used for the analyses. If the two readings differed by more than the previously established set point (0.5 cm for NC), then a third measurement was taken. All three measurements were recorded, and the outlier was excluded during the data cleaning process. Different cut-points of NC were used to classify central obesity in adolescents and adults [8, 33]. NC for adolescent was categorized as abnormal if circumference was > 34.5 cm for boys and > 31.25 for girls [8], whereas for adults the cut-off points for abnormal were > 39 cm for men and > 35 cm women [33]. These cut-points are probably the best to determine individuals with central obesity and metabolic syndrome [8, 33]. The interviewers were trained to collect all measurements by certified nutritionists/dietitians who simultaneously operated as supervisors of the fieldwork.

### Sociodemographic and SEL variables

A questionnaire was used to collect information about demographics such as age (expressed in years), gender, years of education, race/ethnicity, and marital status. SEL was also evaluated by questionnaire using a format based on the national indexes used in each country [34–41]. SEL data were divided into three strata (low, medium, and high). Individuals were asked about sex, age, and education level (equivalent to non-formal education or primary school), middle (equivalent to secondary school), and high (equivalent to technical or university degree). These sociodemographic variables were included as covariates in all statistical models.

#### Statistical analyses

Statistical analyses were carried out using the R software [42]. A Kolmogorov-Smirnov test was applied to evaluate the data distribution. Mean, standard deviation, and frequencies were computed to describe the variables studied.

Multilevel logistic models, including country and region as random effects and adjusted for sex, age, SEL, and educational level, were used to study the association between MVPA (min/day) and NC (dependent variable: normal or elevated values). Multilevel models were carried out using the R package lme4 [43]. A significance level of 5% was set.

## Results

The sample included 2370 participants, aged from 15 to 65 years old (M = 36.6, SD = 14.2), who used the accelerometer, which represented 29.6% of the total sample of the ELANS project (*n* = 9218). There were no significant differences between the participants who used the accelerometer and those who did not concerning sex (*p* = 0.937), SEL (*p* = 0.501), or educational level (*p* = 0.235). However, the distribution by age group was different (*p* = 0.018); the participants with accelerometer were slightly older.

The participants used the accelerometer between 5 and 7 days. On average, the accelerometer was used for 15.3 h/day, which represented a total time of 100.7 h. This time ranged from 14.8 h/day in Costa Rica to 15.8 h/day in Peru (Supplementary material 1).

There were slightly more female (52.2%) than male (47.8%) participants. About 2 out of 3 participants were aged from 20 to 49 years old (65.8%). About half (51.2%) were from low SEL, and more than half (60.1%) had a basic educational level or lower. Only 10.0% were from a high SEL, and 10.2% had a university degree (Table 1).

**Table 1:** Descriptive analysis (%) of sample profile concerning sex, age group, socioeconomic, and educational level of adolescents and adults from eight Latin America countries

Country	N	Sex (%)		Age group (%)				Socioeconomic level (%)			Educational level (%)		
		Male	Female	15–19	20–34	35–49	50–65	Low	Medium	High	Basic or lower	High school	University degree
Argentina	293	44.4	55.6	10.6	30.7	33.1	25.6	53.9	41.3	4.8	73.7	23.9	2.4
Brazil	563	46.2	53.8	11.0	36.2	29.0	23.8	41.7	50.4	7.8	44.9	46.7	8.3
Chile	297	46.5	53.5	10.8	36.7	27.6	24.9	41.4	47.5	11.1	61.6	23.6	14.8
Colombia	339	49.9	50.1	10.0	35.7	27.4	26.8	64.0	31.0	5.0	66.4	22.1	11.5
Costa Rica	273	47.6	52.4	13.6	35.5	30.0	20.9	34.1	54.2	11.7	83.9	11.4	4.8
Ecuador	268	50.4	49.6	13.4	40.3	29.9	16.4	44.8	40.7	14.6	79.1	11.9	9.0
Peru	330	48.2	51.8	14.5	39.7	24.8	20.9	47.0	30.0	23.0	20.6	67.6	11.8
Venezuela	367	49.9	50.1	13.4	42.2	27.8	16.6	80.9	14.2	4.9	69.2	13.1	17.7
Full sample	2730	47.8	52.2	12.1	37.2	28.6	22.2	51.2	38.8	10.0	60.1	29.7	10.2

The average time of MVPA was 34.88 min/day, ranging from 31.16 in Venezuela to 40.27 in Chile. Concerning NC, 37.0% of the full sample was classified as having elevated NC. Chile was the country with the highest percentage of people with elevated NC with more than half (56.9%), and Colombia had the lowest percentage (24.8%) (Table 2).

**Table 2:** Descriptive analysis of moderate-to-vigorous physical activity (Mean and SD) and neck circumference (frequency and %) of adolescents and adults from eight Latin America countries (*n* = 2730)

Country	MVPA	Neck circumference	
	Min/day Mean (SD)	Normal n (%)	Elevated n (%)
Argentina	32.81 (22.63)	169 (57.7%)	124 (42.3%)
Brazil	33.37 (24.63)	405 (71.9%)	158 (28.1%)
Chile	40.27 (23.66)	128 (43.1%)	169 (56.9%)
Colombia	34.84 (24.63)	255 (75.2%)	84 (24.8%)
Costa Rica	32.41 (28.19)	149 (54.6%)	124 (45.4%)
Ecuador	40.04 (30.58)	188 (70.1%)	80 (29.9%)
Peru	36.43 (26.60)	212 (64.2%)	118 (35.8%)
Venezuela	31.16 (21.91)	213 (58.0%)	154 (42.0%)
Full sample	34.88 (25.40)	1719 (63.0%)	1011 (37.0%)

*MVPA* moderate-to-vigorous physical activity

Tables 3 and 4 present the results of the multilevel logistic regression analysis describing the association between MVPA and NC. The effect of daily MVPA (min/day) on NC, considering two hierarchical levels (country and region) and adjusted for sex, age, SEL, and educational level, is presented in Table 3. Overall, the increase of one min/day of MVPA is associated with a decrease of 0.6% in the chance of having an elevated NC (OR = 0.994, CI95% = 0.990–0.998, *p* = 0.003). The association was similar across all countries but only significant in two: Costa Rica (OR = 0.980, CI95% = 0.964–0.997, *p* = 0.024) and Peru (OR = 0.989, CI95% = 0.980–0.999, *p* = 0.031) (Table 4).

**Table 3:** Multilevel logistic models for moderate-to-vigorous physical activity (min/day) with neck circumference of adolescents and adults from eight Latin America countries-full sample (*n* = 2730)

Independent variables	Dependent variable: Neck circumference (normal and elevated)	
	OR (CI95%)	*p*
MVPA (min/day)	0.994 (0.990, 0.998)	0.003
Sex female (vs. male)	0.739 (0.624, 0.875)	< 0.001
Age (years)	1.013 (1.007, 1.019)	< 0.001
Socioeconomic level medium (vs. low)	1.143 (0.947, 1.380)	0.164
Socioeconomic level high (vs. low)	1.003 (0.731, 1.378)	0.983
Educational level high school (vs. basic or lower)	0.756 (0.613, 0.932)	0.009
Educational level university degree (vs. basic or lower)	0.780 (0.573, 1.062)	0.114

Multilevel logistic model with three levels (individual, region, country)

*MVPA* moderate-to-vigorous physical activity, *OR* odds ratio, *CI95* 95% confidence interval

**Table 4:** Multilevel logistic models for moderate-to-vigorous physical activity (min/day) with neck circumference of adolescents and adults from eight Latin America countries-in each country (*n* = 2730)

Countries	Dependent variable Neck circumference (normal and elevated)	
	OR (CI95%)	*p*
MVPA (min/day)		
Argentina	0.996 (0.984, 1.008)	0.490
Brazil	0.990 (0.975, 1.006)	0.214
Chile	0.998 (0.982, 1.015)	0.846
Colombia	0.990 (0.979, 1.002)	0.105
Costa Rica	0.980 (0.964, 0.997)	0.024
Ecuador	0.997 (0.984, 1.010)	0.643
Peru	0.989 (0.980, 0.999)	0.031
Venezuela	0.990 (0.976, 1.004)	0.178

Multilevel logistic models, one for each country, with two levels (individual, region), adjusted for sex, age, socioeconomic level, and educational level

*MVPA* moderate-to-vigorous physical activity, *OR* odds ratio, *CI95* 95% confidence interval

## Discussion

This study aimed to investigate the association of accelerometer-determined MVPA with NC in adolescents and adults from eight LA countries. The average time of MVPA was 34.88 min/day, and 37.0% of the full sample was classified as having elevated NC. Overall, the increase of one min/day of MVPA is associated with a decrease of 0.6% in the chance of having an elevated NC. When analyzed by country, the association was similar across all countries but only significant in Costa Rica and Peru. The effect significance of MVPA (min/day) on NC, considering two hierarchical levels (country and region), was adjusted for sex, age, SEL, and education level.

Latin America is the most urbanized region in the world (80% of Latin Americans live in cities) with demographic and epidemiological changes. Furthermore, high population density, disorganized transit systems, traffic congestion, pronounced income inequality, and important and rapid nutritional transition are some of the characteristics of LA. However, one point stands out: the marked increase in the prevalence of obesity and chronic diseases in nearly all of LA [44–46]. More than 20% of population from LA, including youth are overweight or obese, and the indices has increased to a greater scale specifically in Mexico, Argentina, and Chile. [47] and in 2030, up to 81.9% of the LA adult population could be either overweight or obese [48]. This is the first study to report NC for a representative sample of LA adolescents and adults from an urban setting; therefore, the only possible comparison was against previous reports that used other anthropometric methods. The prevalence of obesity (BMI ≥ 30 kg/m^2^) in São Paulo, Brazil, was more than 23% and in Santiago, Chile, was more than 31% [49]. In our study, we found 28.1% elevated NC in Brazil and 56.7% in Chile.

The prevalence of overweight and obesity is increasing in LA, and high rate of body fat is a risk factor for increase closely associated factors (i.e., chronic disease, hypertension and type II diabetes) [50]. Epidemiologic study have identified each 5 kg/m^2^ higher BMI was associated, on average, with 30% higher overall mortality, specifically 40% for vascular diseases; 60–120% for diabetes; 10% for cancer; and 20% for respiratory diseases and all other causes [51].

NC provide high values in prediction of distribution of upper-body subcutaneous, is valid marker and the data collection is simple to execute [52–56]. BMI and others anthropometric indicators have long been suggested of defining the overweight and obesity of person [57]. Upper body fat distribution has been considered a risk factor of CVD. It has been reported that free fatty acids are released in larger proportion from upper body subcutaneous fat than lower body subcutaneous fat [58]. Moreover, NC has been used as an index for such an adverse risk profile [59, 60]. Joshipura et al. [9] showed that NC was significantly associated with metabolic factors, including components of metabolic syndrome (hypertension, triglycerides, glucose ≥100, HOMA-IR); the magnitude of the associations is modest, ranging from 0.45–0.66. Importantly, compared to traditional anthropometric measures such as BMI, WC, and body fat percent, NC showed higher significant association with prediabetes and with HDL-C, [9].

This is the first study reporting significant association of accelerometer-determined MVPA with NC of nationally representative samples from urban populations from LA countries (Argentina, Brazil, Chile, Colombia, Costa Rica, Ecuador, Peru, and Venezuela). This study supports previous research that has shown negative relationships between accelerometer-determined MVPA and body composition variables [61, 62]. Our results corroborate those of Van Dyck et al. [62], who reported significant associations of accelerometer-determined MVPA with BMI in adults from 12 countries, independent of country and SEL. Our study showed significant association between MVPA and NC, independent of sex, age, SEL, and educational level. Both our study and the Van Dick et al. [62] study used the cut-point of ≥1952 cpm for MVPA [28]. Higher levels of physical activity may have important additional beneficial effects on fitness or other health outcomes [63]. However, one must keep in mind that the present results are cross-sectional; therefore, no true dose–response relationships can be assumed.

Table 4 presents the results of the multi-level logistic regression analysis describing the significant association between MVPA and NC for Costa Rica (OR = 0.980, CI95% = 0.964–0.997) and Peru (OR = 0.989, CI95% = 0.980–0.999), considering two hierarchical levels (country and region) and adjusted for sex, age, SEL, and educational level. One conclusion from these results is that different patterns of MVPA are associated with high prevalence estimates of elevated NC, so countries could tailor physical activity and obesity promotion strategies to local infrastructure, available programs, and culture. The results shows the variety and diversity of countries from LA, and, thus, the need to better understand these realities for to expand healthy eating habits in LA [64, 65]. For example, Peruvian people have increased their BMI during fifteen years (1996–2011), and more than global female trend rate [66]. Worldwide, adult BMI has increased by 0.4–0.5 kg/m^2^ per decade [66]. Moreover, adult obesity levels have been augmented from 6.4 to 12.0% [67]. In addition, sedentary activities and physical inactivity due to rapid urbanization have been contributing to escalating levels of over-nutrition [45]. In a region experiencing escalating rates of obesity and inactivity, there is an opportunity to promote effective interventions as interdisciplinary strategies (i.e., coordination between health, sports and education departments) to increase physical activity, healthy eating habits and prevent noncommunicable diseases.

The results of the study may not be directly generalizable to other countries. Nevertheless, no study has evaluated the accelerometer-determined MVPA and NC of adolescent and adult populations in LA using a standardized methodology across a consortium of several participating countries. The present study had several strengths: the number of participants; comparable data collection protocols; the inclusion of objective data on physical activity makes ELANS a unique Latin American data source. To date, most studies in low- and middle-income countries have had to rely on self-reported physical activity data [68, 69]; and it expands the existing literature by reporting the association of physical activity with NC. However, the ELANS employed a cross-sectional design, the cut-off point of counts/min for classifying the intensity of physical activity (light, moderate and vigorous) was the same for adolescents and adults and to the number of participants that did not have any information on the MVPA.

## Conclusions

When analyzing all countries together, the present study provided evidence of significant associations between MVPA and NC in adolescents and adults from LA, independent of sex, age, SEL, and educational level. When analyzed separately by country, only Costa Rica and Peru showed significant association between MVPA and NC.

This examination of accelerometry data and NC characterizes the primary investigation of these associations in eight LA countries. Further prospective study is need to assess NC can substitute others anthropometrics methods to better elucidate of obesity correlates and to understand the variability observed in urban regions in the detected relations.

## Data Availability

The dataset used and analysed during the current study are available from the corresponding author on reasonable request.

## Abbreviations

BMI: Body mass index
CI95%: 95% Confidence interval
CVD: Cardiovascular disease
ELANS: Latin American Study of Nutrition and Health / *Estudio Latinoamericano de Nutrición y Salud*
LA: Latin America
M: Mean
MVPA: Moderate-to-vigorous physical activity
NC: Neck circumference
OR: Odds ratio
SD: Standard deviation
SEL: Socioeconomic level
WC: Waist circumference
